# Management of Peripheral Cemento-Ossifying Fibroma Associated With Maxillary Anterior Teeth: A Case Report

**DOI:** 10.7759/cureus.48410

**Published:** 2023-11-06

**Authors:** Unnati Shirbhate, Pavan Bajaj, Mrunal Meshram, Khushboo Durge

**Affiliations:** 1 Department of Periodontics, Sharad Pawar Dental College and Hospital, Datta Meghe Institute of Higher Education and Research (Deemed to be University), Wardha, IND; 2 Department of Oral Medicine and Radiology, Sharad Pawar Dental College and Hospital, Datta Meghe Institute of Higher Education and Research (Deemed to be University), Wardha, IND

**Keywords:** fibroma, scalpel, histopathology, surgical, management, peripheral cemento-ossifying fibroma

## Abstract

The peripheral cemento-ossifying fibroma (PCOF) lesion primarily affects females in their second decade of living. These lesions are more frequently associated with the gingival margin, the anterior surface of the molars, and the maxilla. On clinical examination, PCOF typically appears as a well-differentiated, slowly expanding gingival mass in the interdental papilla region that is less than 2 cm in size. The surface may seem ulcerated, the base may be sessile or sometimes pedunculated, and the colour is either the same as the gingiva or reddish. The histological examination, which identifies cellular connective tissue and the focal presence of bone or calcifications, provides the basis for the final diagnosis. Treatment modalities for the PCOF include surgical excision of the lesion. A 38-year-old female reported slow-growing swelling associated with the maxillary anterior region. Removal of the lesion is done by using a scalpel, and histopathological examination revealed the peripheral type of cemento-ossifying fibroma. This case report demonstrates the management of PCOF lesions with the conventional scalpel approach with the help of proper clinical examination, radiological findings, and histopathological examination, which reveals favourable outcomes in the patient regarding esthetics and improves mastication-related issues and speech.

## Introduction

A benign tumour called osseous fibroma mostly develops in the craniofacial bones. It comprises proliferating fibroblasts that produce osseous products, such as oval calcifications and bone, and these lesions are clearly distinguished from the surrounding bone. Ossifying fibromas can be classified as either central or peripheral. The medullary cavity enlarges in the central type, originating from the endosteum or periodontal ligament adjacent to the root apex. Only the soft tissues covering the jaws' tooth-bearing regions are affected by the peripheral type [1]. One of the primary neoplasms of the periodontium and bone is the cemento-ossifying fibroma. The pathogenesis of this tumour is not known. Some peripheral cemento-ossifying fibromas (PCOFs) are thought to exhibit fibrous development and subsequent calcification due to their clinical and histological similarities. Because it is challenging to diagnose PCOF just from clinical observation, radiographs and a histological study are necessary for a precise diagnosis. To avoid recurrence, the lesion must also be removed entirely [2]. Cemento-ossifying fibroma is the term used by the World Health Organization in 1992 to refer to a combination of two histologic types, ossifying fibroma and cementifying fibroma, which may be having clinically and radiographically indistinguishable characteristics [3].

PCOF is a rare lesion with variable morphologic features. It is described as a well-defined, sometimes encapsulated growth or lesion made of fibrous tissue with different concentrations of mineralised material that resembles either cementum (cementifying fibroma) or bone (ossifying fibroma) [2]. PCOFs most commonly comprise 3.1% of all oral tumours and 9.6% of all gingival lesions. This tumour's pathophysiology is unknown. Certain PCOFs are assumed to go through fibrous maturation and calcification due to their similarities in histology and clinical features. PCOF is frequently linked to irritants such as calculus deposits, bacteria on the plaque, orthodontic bands and brackets, poorly fitting crowns, and uneven restorations. The periodontal ligament or periosteal cells are a probable source of the mineralised product [2,4]. Radiographs typically show no apparent underlying bone involvement. On the other hand, there are instances of noticeable superficial bone degradation. PCOF should be surgically removed and examined for histopathology to confirm the diagnosis. A recurrence rate of PCOF with 8-20% has been reported [5].

## Case presentation

**Figure 1:**
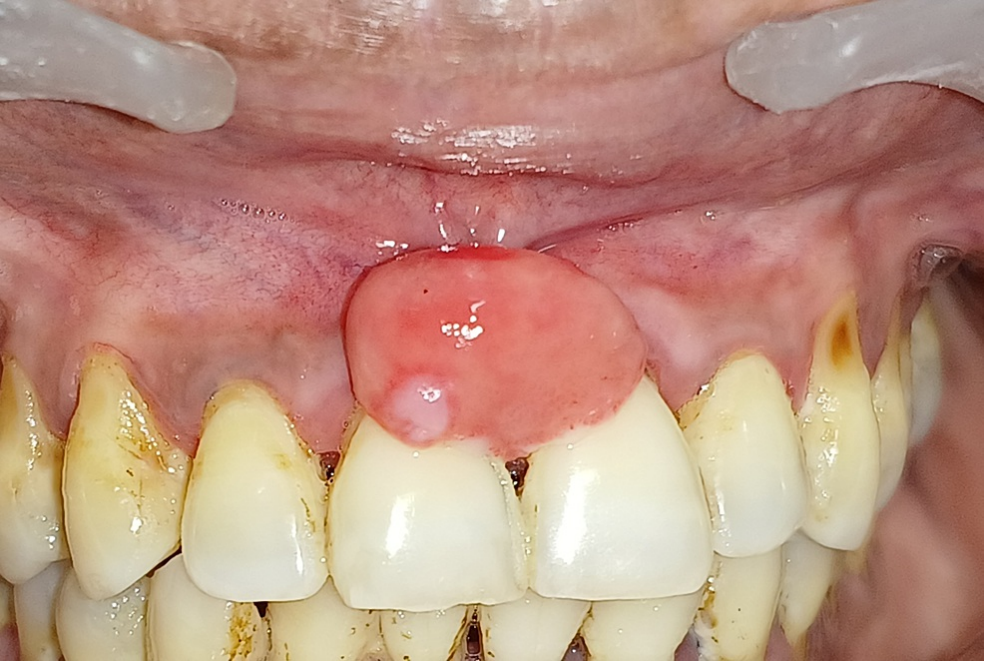
Preoperative view of peripheral cemento-ossifying fibroma

A 38-year-old female with a chief complaint of a slow-growing, painless soft tissue growth between her maxillary anterior teeth reported to the outpatient department (OPD) of periodontics. Almost a year ago, the lesion began as a little papule. The patient had a history of difficulty speaking and chewing. A pedunculated, firm, non-tender growth on the papillary and marginal gingiva in connection to the maxillary right and left central incisor was found during the clinical examination shown in Figure 1.

**Figure 2:**
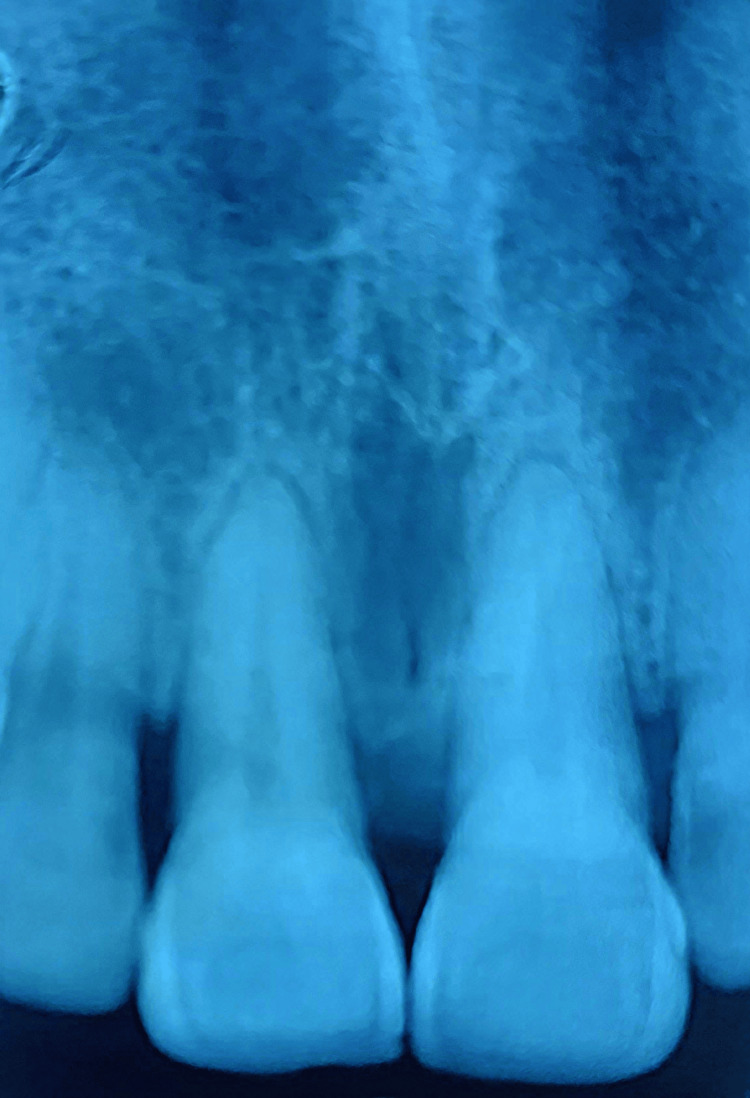
Intraoperative radiograph showing radiopaque areas suggestive of calcification between the maxillary central incisors

Initially, the differential diagnoses were irritational fibroma, PCOF, pyogenic granuloma, and peripheral giant cell granuloma. As seen in Figure 2, an intraoral periapical (IOPA) radiograph showed soft tissue shadows, interspersed with radiopacity suggestive of calcification at the site of the lesion between the maxillary central incisors, with regard to that of exophytic growth.

**Figure 3:**
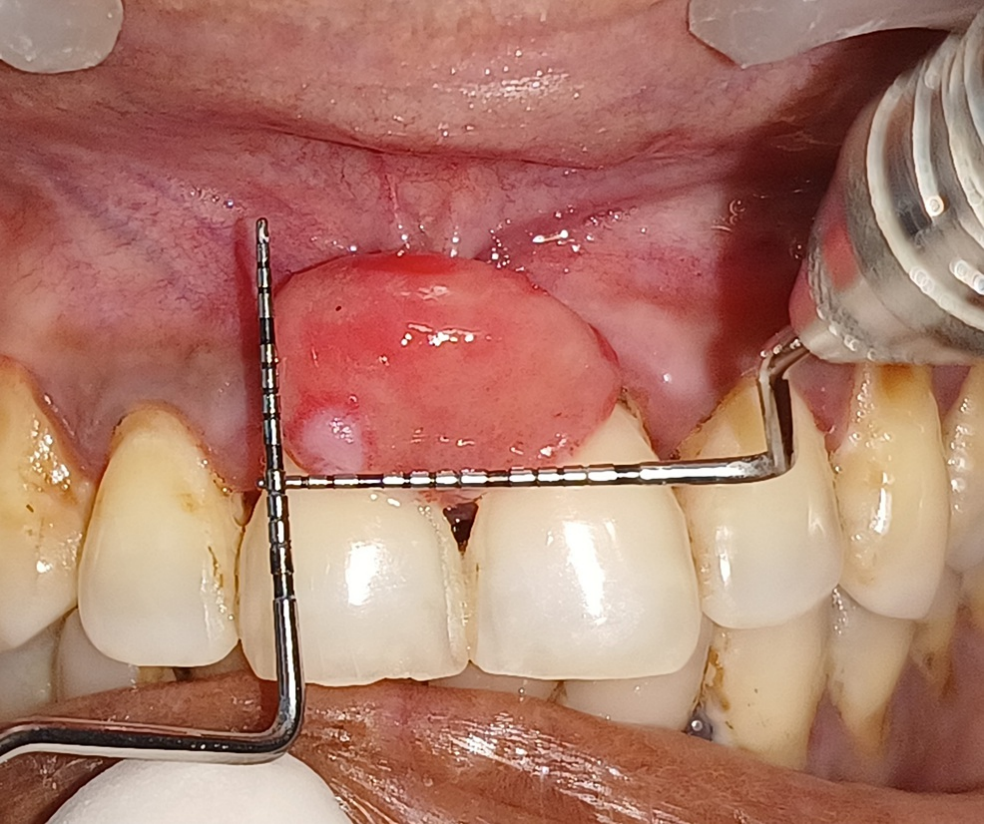
Measurements at the site of PCOF lesion taken which were 10-15 mm long and wide, respectively

Measurements were taken, which revealed a growth size of 10-15 mm long and wide, as seen in Figure 3.

**Figure 4:**
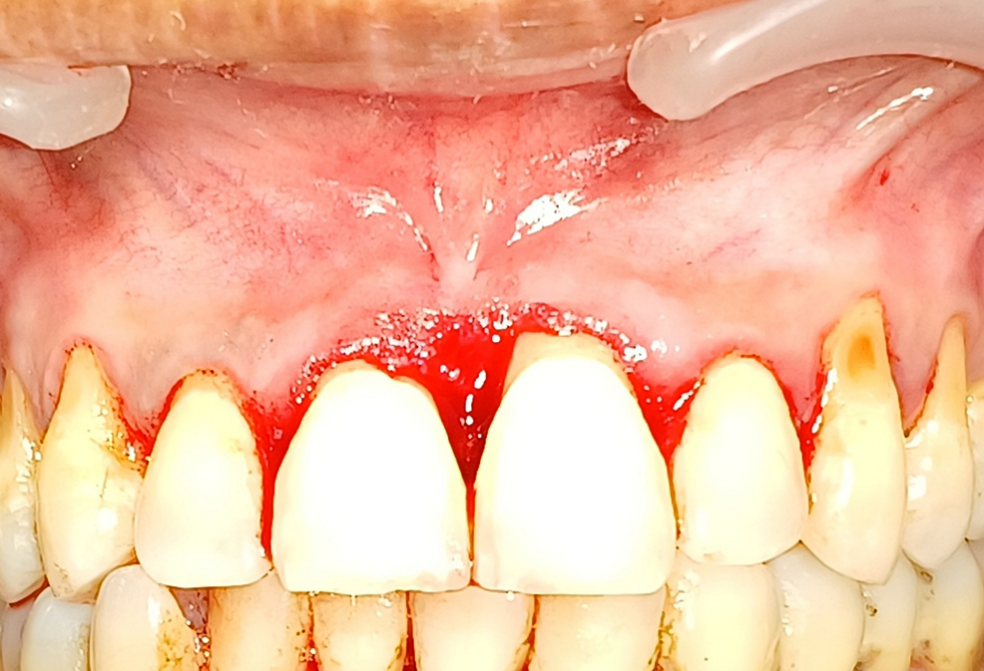
Surgical excision of peripheral cemento-ossifying fibroma done

The patient had a history of poor oral hygiene. Considering the above findings, the patient was advised for initial therapy, surgical therapy, and haematological examination. The scaling and polishing were done before surgical treatment. Haematological analysis revealed investigation within normal range. Informed written consent was taken from the patient. Surgical intervention was carried out with the conventional scalpel technique, shown in Figure 4, where the soft tissue growth was excised and haemostasis was achieved by ligating vessels and direct pressure with packs and gauzes.

**Figure 5:**
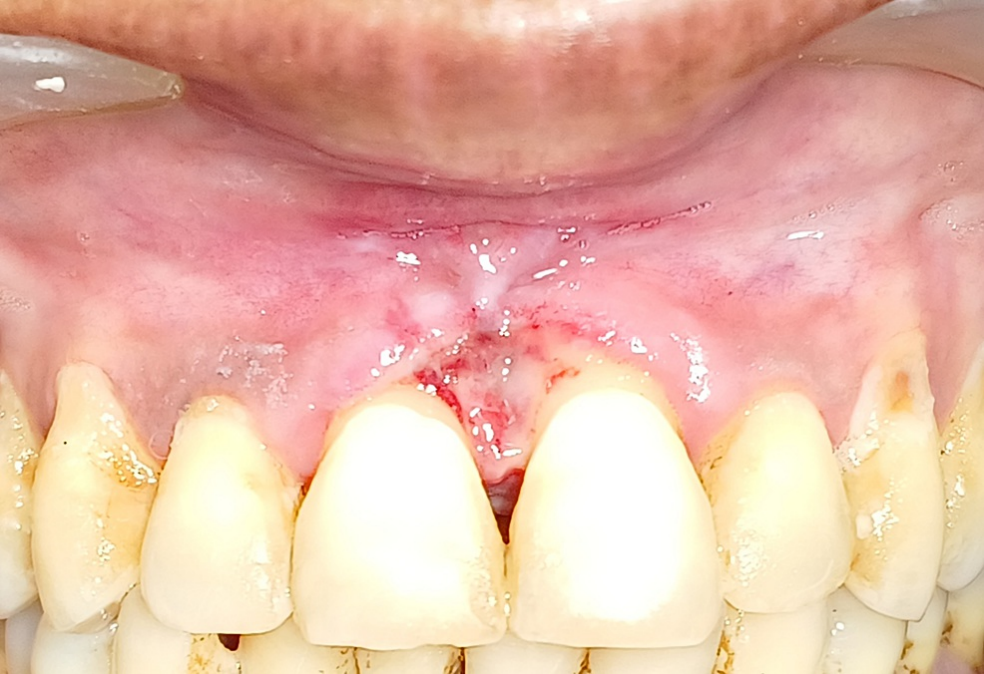
Postoperative view after seven days showing satisfactory healing of the lesion

Postoperative instructions and medications were prescribed, and the patient was reviewed after seven days of surgery; on examination, the lesion was found to be healing satisfactorily as seen in Figure 5.

**Figure 6:**
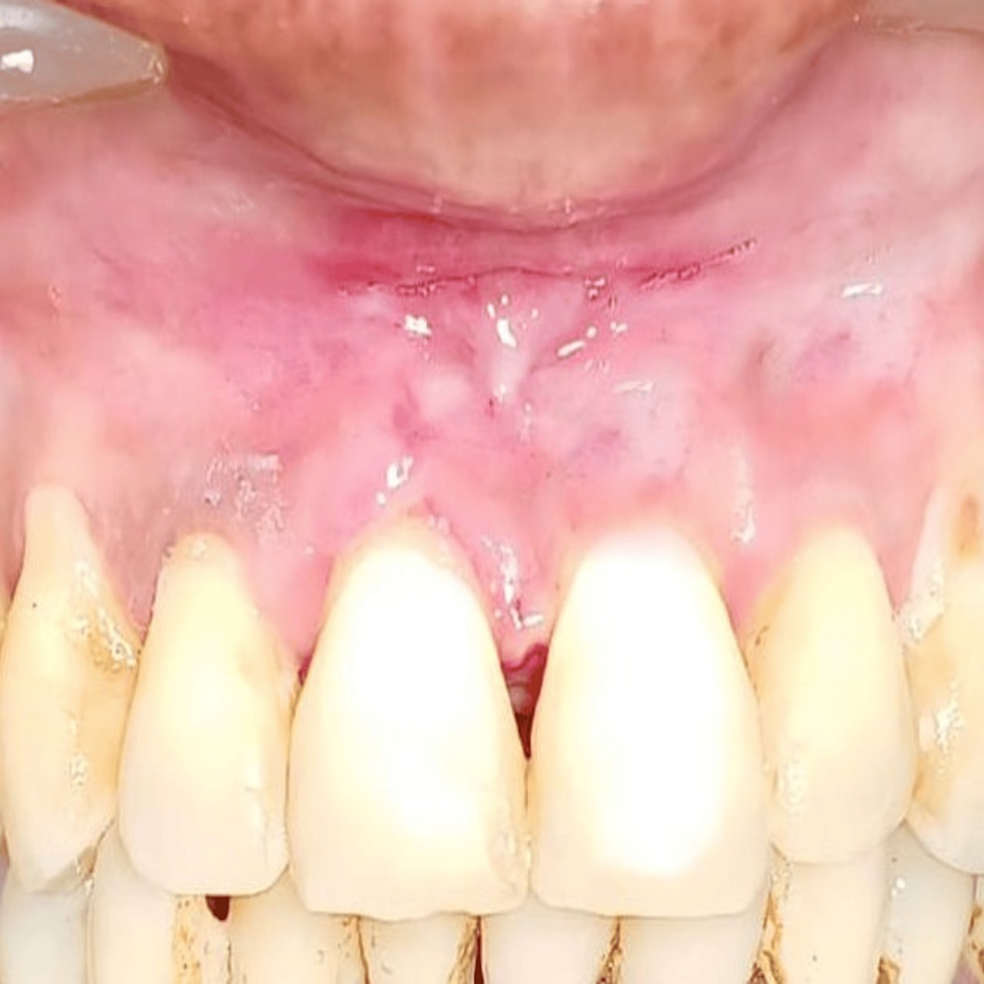
Postoperative view after 15 days showing complete satisfactory healing and improved esthetics

After 15 days of surgical intervention, a follow-up examination revealed complete wound healing and no discomfort and pain and improved esthetics at the operated site, which can be appreciated in Figure 6. The patient was reviewed for the next three and six months and revealed no recurrence and discomfort.

**Figure 7:**
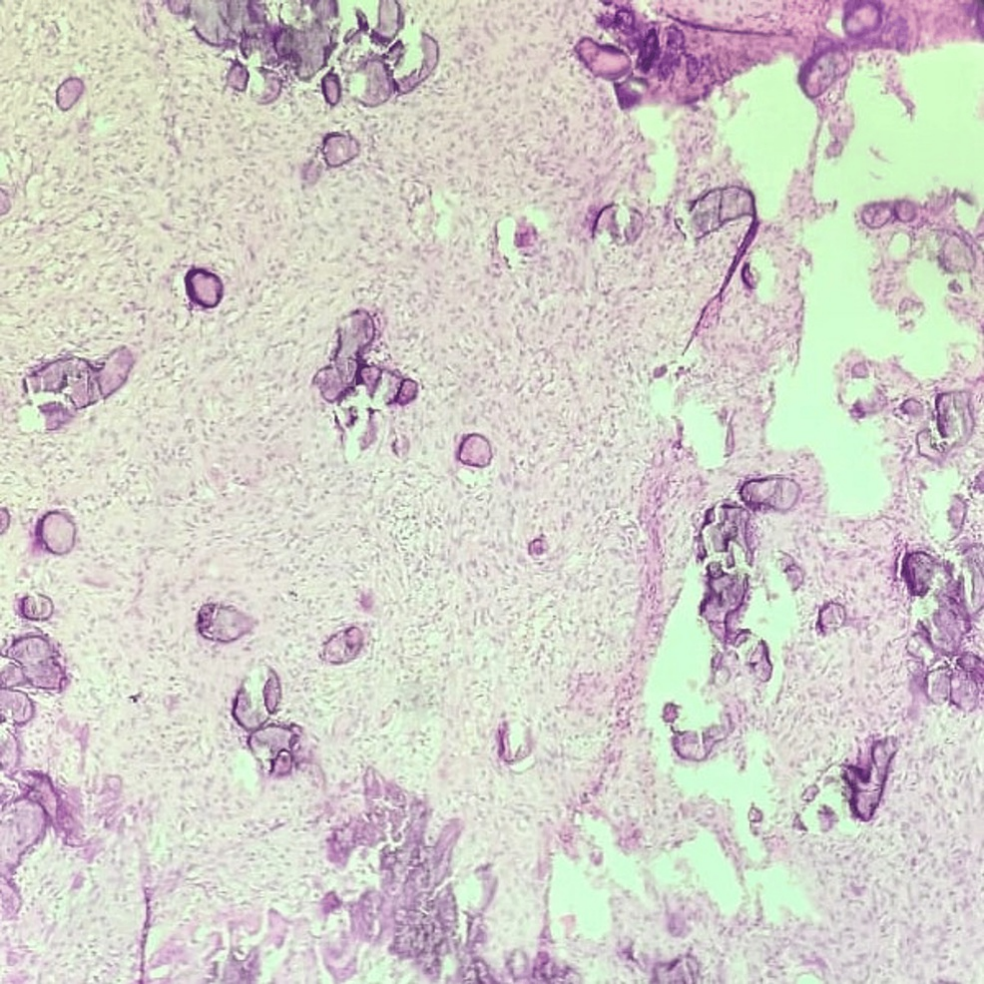
Histopathological examination showing stratified squamous epithelium covering a highly cellular mass of connective tissue

The excised specimen after the surgery was sent for histopathological examination, which confirmed the diagnosis of PCOF, as illustrated in Figure 7. The lesion displays stratified squamous epithelium covering a highly cellular mass of connective tissue composed of areas of mineralisation, fibroblasts, fibrocytes, and fibrillar stroma.

## Discussion

A clearly defined and sometimes enclosed lesion is called a PCOF. Fibrous tissue with varying calcific material levels that resemble cementum (cementifying fibroma) or bone (ossifying fibroma) makes up this condition. The exact cause of PCOF remains unknown, but it has been proposed that periodontal ligament cells are the source of these lesions [6]. The occurrence of PCOF is mostly common in women, suggesting that hormone influences may be involved. It has a high incidence in the second and third decades. Approximately 60% of tumours involve the maxilla, most often the anterior gingiva. Regarding clinical manifestation, PCOF is a slowly developing gingival growth that is less than 2 cm in size and is typically found in the interdental gingiva [5,7].

Based on the focal presence of bone or other calcifications in cellular connective tissue, a histological examination is essential for a precise diagnosis. This highlights the difficulty in diagnosing PCOF based solely on clinical observations, underscoring the requirement for the histological evaluation of biopsy specimens for a correct diagnosis [8]. The predominant regions of highly cellular connective tissue with foci of mineralisation in the form of bone, cementum-like material, and a dystrophic type of calcification are PCOF's most distinctive histological features [9]. Treatment for PCOF entails managing all irritating factors that may lead to a recurrence, along with the complete removal of the lesion, including the periosteum and periodontal ligament, due to its high recurring rates [8,10].

## Conclusions

This case report demonstrates that surgical excision is a simple, efficient, and affordable method for treating PCOF and giving aesthetic and functional advantages to the patient in terms of mastication and chewing. Surgical intervention and long-term follow-up results showed favourable outcomes regarding issues related to mastication and speech.
